# Nano-Enabled Seed Treatment Using Bisepoxide-Polyoxypropylenetriamine Polymeric Gel with Different Embedded Zinc Sources

**DOI:** 10.3390/gels11030167

**Published:** 2025-02-26

**Authors:** Felipe B. Alves, Adela S. M. Goñi, Bruno A. Fico, Vanessa S. A. Silva, Renato P. Orenha, Renato L. T. Parreira, Heber E. Andrada, Gabriel Sgarbiero Montanha, Higor J. F. A. da Silva, Eduardo de Almeida, Hudson W. P. de Carvalho, Natália Chittolina, Clíssia B. Mastrangelo, Eduardo F. Molina

**Affiliations:** 1 University of Franca, Av. Dr. Armando Salles Oliveira 201, Franca 14404-600, SP, Brazil; felipeb.alves@hotmail.com (F.B.A.); adelasanmartin64@gmail.com (A.S.M.G.); bruno.fico@outlook.com (B.A.F.); vanessasilveira3012@gmail.com (V.S.A.S.); rpo9@hotmail.com (R.P.O.); renato.parreira@unifran.edu.br (R.L.T.P.); heberandrada@hotmail.es (H.E.A.); 2 Public University of Navarre, Arrosadia Campus, Av. Cataluña, 31006 Pamplona, Navarre, Spain; 3 Center for Nuclear Energy in Agriculture (CENA), University of São Paulo, Piracicaba 13416-000, SP, Brazil; gabriel.montanha@usp.br (G.S.M.); higorjfas@usp.br (H.J.F.A.d.S.); edualm@cena.usp.br (E.d.A.); hudson.carvalho@um6p.ma (H.W.P.d.C.); nchittolina@usp.br (N.C.); clissia@usp.br (C.B.M.); 4 Mohammed VI Polytechnic University, Lot 660, Ben Guerir 43150, Morocco

**Keywords:** seed priming, amine–epoxide, zinc-based fertilizers, nutrient distribution

## Abstract

In the 21st century, sustainable agriculture is expected to become a major contributor to food security and improved nutrition. Amine–epoxide-based materials have great potential for use in agriculture due to their tunable physicochemical features, which are dependent on the concentration and composition of the monomers. In this work, catalyst-free green synthesis, using only water as a solvent, was performed to obtain a nanocarrier (TGel) capable of transporting nutrients after seed priming. The synthesis was based on the opening of the epoxy ring by nucleophile attack, using an amine-terminated polyether. Transmission electron microscopy (TEM) and dynamic light scattering (DLS) techniques showed the spherical morphology of the particles, which ranged in size from 80 nm (unloaded TGel) to 360 nm (zinc-loaded TGel), respectively. Theoretical bonding analysis revealed that Zn cation species from the ZnSO_4_ source interact with the polymer via σ-bonds, whereas EDTA forms hydrogen bonds with the polymer, thereby enhancing noncovalent interactions. Micro X-ray fluorescence (μ-XRF) and energy-dispersive X-ray fluorescence spectroscopy (EDXRF) provided details of the distributions of Zn in the seed compartments and shoots of cucumber plants after seed priming and plant growth, respectively. The use of the Zn-loaded TGels did not affect the physiology of the cucumber plants, as indicated by the photosynthetic efficacy, chlorophyll, and anthocyanin indices.

## 1. Introduction

The modern agricultural system encounters numerous challenges originating from the impacts of global climate change, soil degradation, pest and disease, and the inefficient use of agrochemicals, which significantly hinder agricultural productivity. Addressing these challenges requires the development and implementation of innovative agricultural strategies to improve efficiency and promote long-term sustainability [1]. Seed priming with nanomaterials offers a promising approach by improving seed protection during storage, enhancing germination rates and uniformity, and supporting early plant development [2]. In the last decade, there have been significant advances in the development and application of polymeric materials (natural, synthetic, and/or functionalized polymers) in agriculture and the food industry [3,4]. This progress has been primarily driven by the growing demand for food, as well as concerns about food safety. These factors highlight the need for increased efficiency in crop cultivation, food production, and processing methods to achieve high yields, improved quality, and mitigation of potential health risks associated with food consumption [5,6]. The advantageous properties exhibited by polymers (considering their physical form, porosity, permeability, diffusion capacity, chemical reactivity, and stability) make them highly suitable for use in the agricultural area [7,8], where their integration with nano-engineering systems can assist in addressing key issues related to health standards, nutritional enrichment, environmental conservation, pollution mitigation, and economic growth. Consequently, the utilization of polymers in agriculture and the food industry is a dynamic and expanding theme that can contribute to meeting emerging demands and challenges in various sectors essential for human sustenance and well-being [9].

The specific properties of a polymeric material can be tailored based on fundamental considerations, involving two critical stages: (i) the selection of appropriate monomers to achieve the desired arrangement of structural constituents within macromolecules and (ii) the application of optimized polymerization techniques [10,11]. Polymers have diverse applications within the agricultural sector, including (but not limited to) soil conditioning, formulation of planting and transplantation gels, seed coatings to regulate germination, soil aeration, and soil sterilization [12,13,14,15].

Micro/nanosized polymeric particles have many uses in the areas of chemicals and materials [16,17]. The development of methods for the fabrication of polymeric particles with adjustable properties is highly desirable for obtaining controlled-release formulations of various agrochemicals, which can reduce the amounts of these chemicals released to the environment [18]. Therefore, this study investigated the formation of an epoxide monomer from diepoxy poly(ethylene glycol) (DPEG) and an amine from a PPG-based polyetheramine of the Jeffamine T-series to produce amine–epoxide particles. Jeffamine T-series is a polyetheramine characterized by a backbone composed of repeating oxypropylene units. The amine groups are positioned on secondary carbon atoms at the end of aliphatic polyether chains. The “click” reaction was used to obtain the polymeric systems (micro- or nanoparticles), using a catalyst-free chemical reaction that formed hydroxyl groups after the reaction of the epoxide with the amine from the polyoxypropylene backbone [19]. Subsequently, the polymeric amine–epoxide formulation was loaded with different Zn sources (ZnSO_4_ or Zn-EDTA) to study the potential of the materials for use in seed priming applications as carriers for nutrients and other substances essential for plant development. These Zn sources were selected because they are commonly used as fertilizers and are generally in soluble forms. Zn is an essential micronutrient for plant nutrition, playing a critical role in various physiological and biochemical processes. Ethylenediaminetetraacetic acid (EDTA) is a widely used chelating agent in agriculture. This is the first time that Zn-loaded amine–epoxide particles have been investigated as carriers for applications in agriculture as a new seed priming technology.

One of the most effective strategies for enhancing plant development, growth, and yield is seed treatment. This cost-effective and straightforward technique induces a priming effect, could lead to a strengthening seedling vigor, and enhances germination rate, biomass accumulation, crop productivity, and the maintenance of ionic homeostasis [16]. This work demonstrated the potential of the technique by using Zn-loaded amine–epoxide polymeric particles to improve the absorption of micronutrients by the different seed compartments without any adverse effects on plant growth. After initial seed priming with the Zn-loaded polymeric formulations, the physiological parameters of cucumber seedlings were evaluated during plant growth over a period of 18 days.

## 2. Results and Discussion

### 2.1. Characterization of the Zn-Loaded and Unloaded TGels

**Figure 1:**
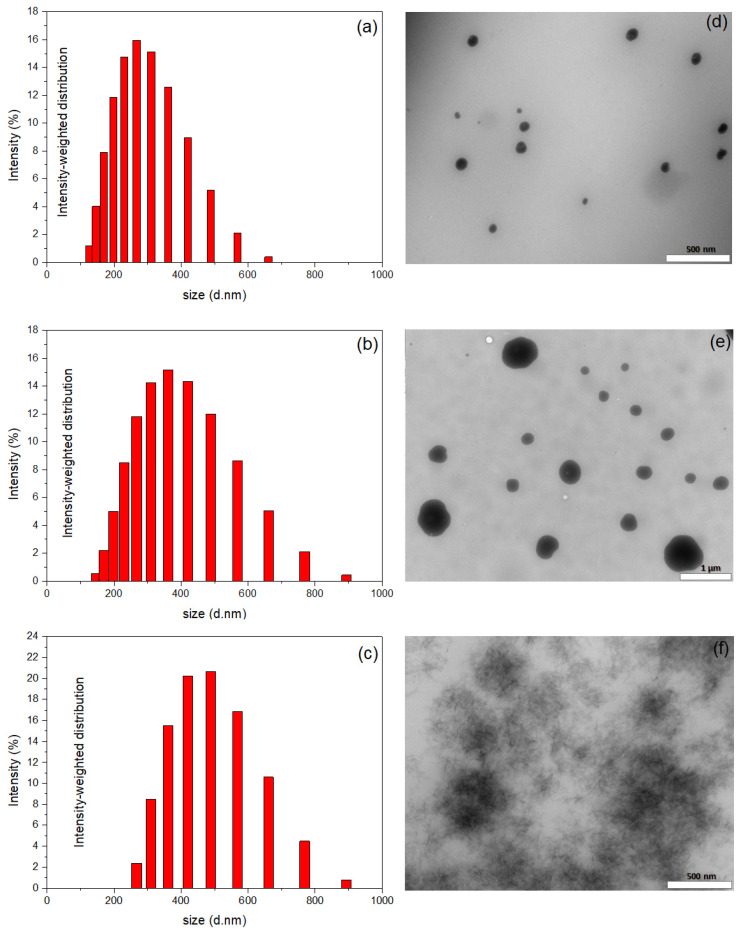
Size distributions obtained by DLS for (**a**) pure TGel, (**b**) TGel-ZnEDTA, and (**c**) TGel-ZnSO_4_. TEM images of (**d**) pure TGel, (**e**) TGel-ZnEDTA, and (**f**) TGel-ZnSO_4_. The scale bars in the images correspond to 500 nm (**d**,**f**) and 1 μm (**e**).

The synthesized TGels, both unloaded and loaded with Zn, were thoroughly characterized using DLS and TEM techniques (Figure 1). The unloaded TGel particles had a mean diameter of ~267 nm and polydispersity index (PdI) of 0.136 (Figure 1a). At pH 7, the zeta potential (ζ) of the TGel was +34 mV. When the zinc sources were added, the diameters observed by DLS were ~361 nm for TGel-ZnEDTA (with PdI of 0.110) and ~489 nm for TGel-ZnSO_4_ (with PdI of 0.225), as shown in Figure 1b and Figure 1c, respectively. The zeta potentials of the loaded TGel-ZnEDTA and TGel-ZnSO_4_ decreased, compared with the unloaded TGel, with ζ values (at pH 7) of +15 mV and +18 mV, respectively. Transmission electron microscopy showed that the unloaded TGel particles presented spherical-like morphology, with an average size of 80 ± 5 nm (Figure 1d). The discrepancy between this size and the value obtained by DLS could be explained by the hydration of the TGel in an aqueous environment. Similarly, TGel-ZnEDTA particles exhibited a spherical morphology, comprising both smaller and larger particles, with the latter suggesting an agglomeration (adsorption process) of the former particles by interactions of TGel, EDTA ligand, and Zn^2+^ ions. The average particle size ranged from 180 to 600 ± 20 nm (Figure 1e). Notably, the TGel-ZnSO_4_ showed a distinct morphology (Figure 1f), with the apparent formation of polymer clusters. This different morphology could have been due to the presence of ions (such as Zn^2+^ and SO_4_^2−^) in the solution, which induced the formation of polymer clusters when using ZnSO_4_ embedded in the polymeric gel. These results indicated that the final morphology of the TGel was determined by the nature of the Zn source embedded in the polymeric network. The loaded TGels containing Zn-EDTA and ZnSO_4_ were subsequently used as seed priming agents, aiming at agricultural applications.

**Figure 2:**
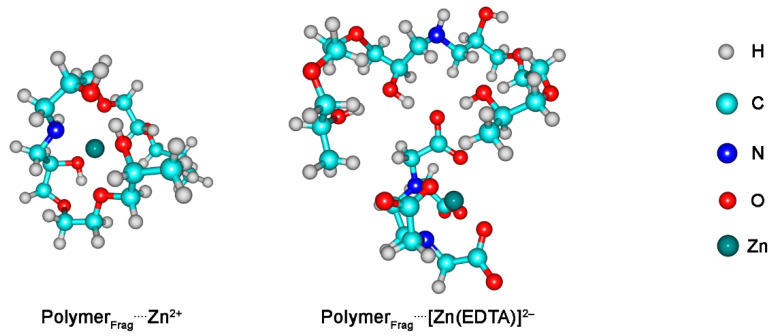
Optimized geometries of the analyzed complexes: Polymer_Frag_····Zn^2+^ and Polymer_Frag_····[Zn(EDTA)]^2−^.

Theoretical calculations were performed to explore the primary chemical interactions between a molecule based on the TGel framework and either the Zn^2+^ cation or the [Zn(EDTA)]^2−^ complex (Figure 2). The EDA method was used to elucidate the mechanisms of bonding between these structures (Table 1). For the Polymer_Frag_····[Zn(EDTA)]^2−^ complex, two chemical bonding analyses were conducted. The first focused on the interaction between the [Polymer_Frag_(EDTA)]^4−^ complex and the Zn^2+^ cation, while the second examined the chemical interaction between Polymer_Frag_ and [Zn(EDTA)]^2−^. In the EDA method [20], the interaction energy, Δ*E*_int_, is decomposed into four main components, as shown below:

Δ*E*_int_ = Δ*V*_elstat_ + Δ*E*_Pauli_ + Δ*E*_oi_ + Δ*E*_disp_ (1)

In Equation (1), the electrostatic energy term (Δ*V*_elstat_) represents classical electrostatic interactions between the unaltered charge distributions of the interacting fragments. The Pauli repulsion term (Δ*E*_Pauli_) accounts for destabilizing interactions between occupied orbitals, which are linked to steric effects. The orbital interaction energy (Δ*E*_oi_) encompasses charge transfer (interactions between occupied orbitals of one fragment and unoccupied orbitals of another) and polarization (the mixing of occupied and unoccupied orbitals induced by the presence of the other fragment). Lastly, the Δ*E*_disp_ term incorporates dispersion corrections, as introduced by Grimme and colleagues [21].

In the Polymer_Frag_····Zn^2+^ interaction, the contributions from Δ*V*_elstat_ (48%) and Δ*E*_oi_ (49%) were comparable, while Δ*E*_disp_ accounted for a smaller proportion (3%) of the total stabilization energy (Δ*V*_elstat_ + Δ*E*_oi_ + Δ*E*_disp_). These findings were indicative of a partially covalent character of the Polymer_Frag_····Zn^2+^ bond. The attractive Δ*E*_int_ energy, supporting the interaction between polymer fragment and Zn^2+^ ion, predominantly arose from the favorable Δ*V*_elstat_, Δ*E*_oi_, and Δ*E*_disp_ components, which collectively overcame the destabilizing Δ*E*_Pauli_ term (Table 1).

The attractive [Polymer_Frag_(EDTA)]^4−^····Zn^2+^ interaction was predominantly driven by Δ*V*_elstat_ (74%), with smaller contributions from Δ*E*_oi_ (25%) and Δ*E*_disp_ (1%) to the total stabilization energy (Δ*V*_elstat_ + Δ*E*_oi_ + Δ*E*_disp_). This suggested that the [Polymer_Frag_(EDTA)]^4−^····Zn^2+^ interaction was primarily noncovalent in nature. Similarly, the favorable Polymer_Frag_····[Zn(EDTA)]^2−^ interaction exhibited a dominant Δ*V*_elstat_ contribution (52%), together with a significant role of Δ*E*_oi_ (36%) and a smaller influence of Δ*E*_disp_ (11%) in the total attractive energy (Δ*V*_elstat_ + Δ*E*_oi_ + Δ*E*_disp_). These results were also indicative of the largely noncovalent character of the Polymer_Frag_····[Zn(EDTA)]^2−^ bond.

The Natural Orbitals for Chemical Valence (NOCV) method provides valuable insights into important orbital interactions, such as those between Polymer_Frag_ and Zn^2+^, by decomposing the interaction into pairwise contributions from the most significant molecular orbitals. Each pairwise orbital interaction within a given chemical bond can be visualized by means of deformation density channels, Δ*ρ*_k_(r), where red regions represent electron density loss, while blue regions indicate electron density gain. Additionally, the NOCV method quantifies the energetic contribution (Δ*E*_oi,k_) of each deformation density channel (Δ*ρ*_k_) to the total orbital interaction energy (Δ*E*_oi_) [22].

**Figure 3:**
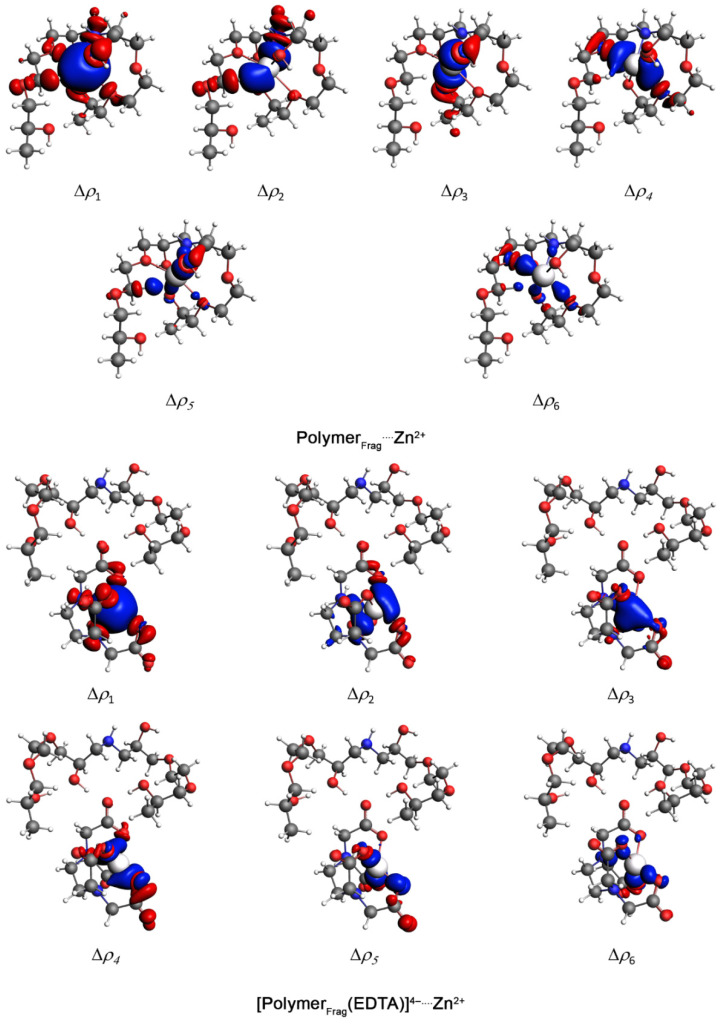
Surface plots of the first six density deformation channels, Δ_ρ1–6_, with an isovalue of 0.001 a.u., for the Polymer_Frag_····Zn^2+^ and [Polymer_Frag_(EDTA)]^4−^····Zn^2+^ complexes. Atom color coding: hydrogen (white), carbon (gray), nitrogen (blue), oxygen (red), and zinc (silver).

**Figure 4:**
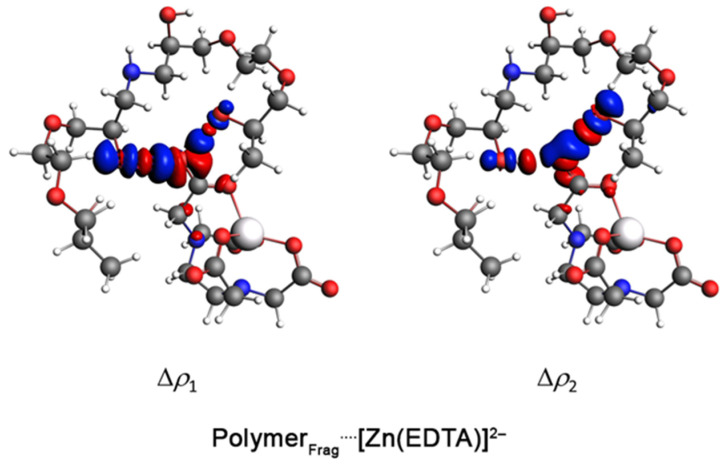
Surface plots of the first two density deformation channels, Δ*ρ*_1_ and Δ*ρ*_2_, with an isovalue of 0.001 a.u for the Polymer_Frag_····[Zn(EDTA)]^2−^ compound. Atom color coding: hydrogen (white), carbon (gray), nitrogen (blue), oxygen (red), and zinc (silver).

The key density deformation channels, Δ*ρ*_1–6_, are associated with the Polymer_Frag_····Zn^2+^ chemical bonds are shown in Figure 3. These results suggested that the interaction between the polymer fragment and the Zn^2+^ ion occurred primarily through the nitrogen and (especially) the oxygen atoms of the polymer, involving σ-bonds. In the case of the interaction between [Polymer_Frag_(EDTA)]^4−^ and Zn^2+^, the NOCV methodology revealed that the Zn^2+^ cation interacted with the oxygen atoms in the EDTA structure by means of σ-bonds (Figure 3). The NOCV analysis of the main density deformation channels, Δ*ρ*_1_ and Δ*ρ*_2_, related to the Polymer_Frag_····[Zn(EDTA)]^2−^ bond showed that the polymer fragment in the Polymer_Frag_····[Zn(EDTA)]^2−^ complex interacted with the EDTA compound by O–H····O hydrogen bonds (Figure 4). The energies associated with the Δ*ρ*_1–6_ channels, Δ*E*_oi,1–6_, are summarized in Table 1.

Theoretical bonding analysis allows us to establish the following correlation. On the one hand, an analysis of the polymer framework and Zn^2^⁺ cation indicates that the coordination of this ionic species with the polymer framework occurs via σ-bonds. Since these results (optimized geometries) only consider the Polymer_Frag_····Zn^2^⁺ interaction, the presence of anionic species ${\mathrm{SO}}_{4}^{2-}$ (originating from the ZnSO_4_ source) may also contribute to interactions with the hydrogen from amine (NH) present at the interface of water–polymer gel, leading to a decrease in ζ values from +34 mV (pure TGel) to +15 mV (TGel-ZnSO_4_). Furthermore, the partial covalent character of the Polymer_Frag_····Zn^2^⁺ bond, along with the possible interaction of SO_4_^2^⁻ with the NH group of the TGel interface, could hinder the formation of spherical particles, as demonstrated in Figure 1c. On the other hand, due to the hydrogen bonds formed by the EDTA compound (O–H····O) with the TGel polymer—primarily through noncovalent interactions—and the Zn^2^⁺ cation maintaining interactions with the oxygen atoms of EDTA (see Figure 3), these features suggest that the EDTA ligand cannot diffuse into polymeric nanoparticles but is instead adsorbed onto the surface of TGel spherical particles. This mechanism strongly aligns with the results from DLS, ζ-potential analysis, and TEM image, which show that the incorporation of ZnEDTA into TGel particles leads to an increase in particle size (see Figure 1b), a decrease in ζ-potential, and the presence of larger, deformed spherical TGel-ZnEDTA particles (Figure 1e).

### 2.2. TGels Containing Different Zn Sources as Safe Seed Nanopriming Agents

**Figure 5:**
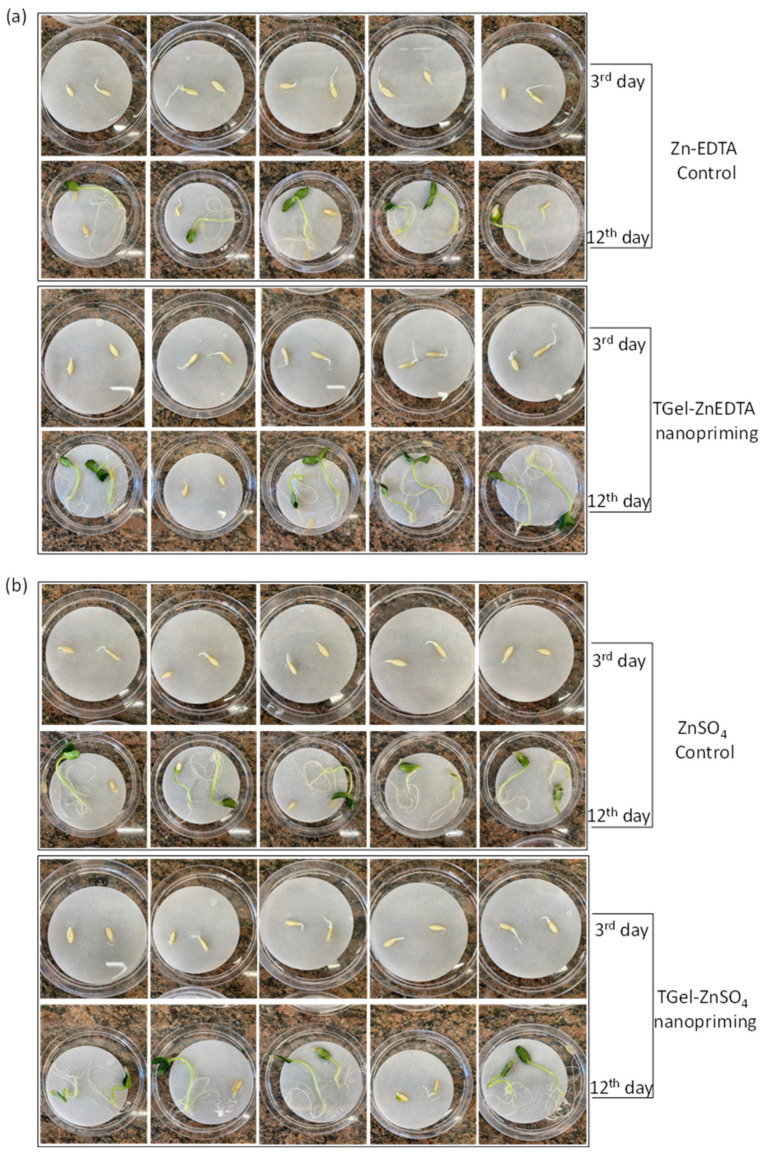
Comparison of cucumber seedling growth at 3rd and 12th days after priming with (**a**) ZnEDTA solution (controls) and TGels containing ZnEDTA source; (**b**) ZnSO_4_ solutions (controls); and after nanopriming with TGels containing ZnSO_4_ source. The images show five replicates of the assays. The germination assays showed two seeds per petri dish with replicates *n* = 5.

The effect of seed nanopriming on the growth of cucumber plants was evaluated by treating seeds with the TGels containing the different Zn sources (Zn-EDTA and ZnSO_4_) and measuring the lengths of roots and shoots during a 12-day period following germination. For comparison, Zn-EDTA and ZnSO_4_ solutions were employed as control seed priming treatments. In these experiments, day 1 was designated as the day that the seeds were placed in Petri dishes after priming. For all the seed treatments, the germination rate exceeded 85%. At a macroscopic level, no phytotoxic effects were observed following the application of the Zn-loaded TGels as nanopriming agents, as evidenced by the germination progression shown in Figure 5, comparing the Zn-EDTA control, TGel-ZnEDTA, ZnSO_4_ control, and TGel-ZnSO_4_ treatments.

**Figure 6:**
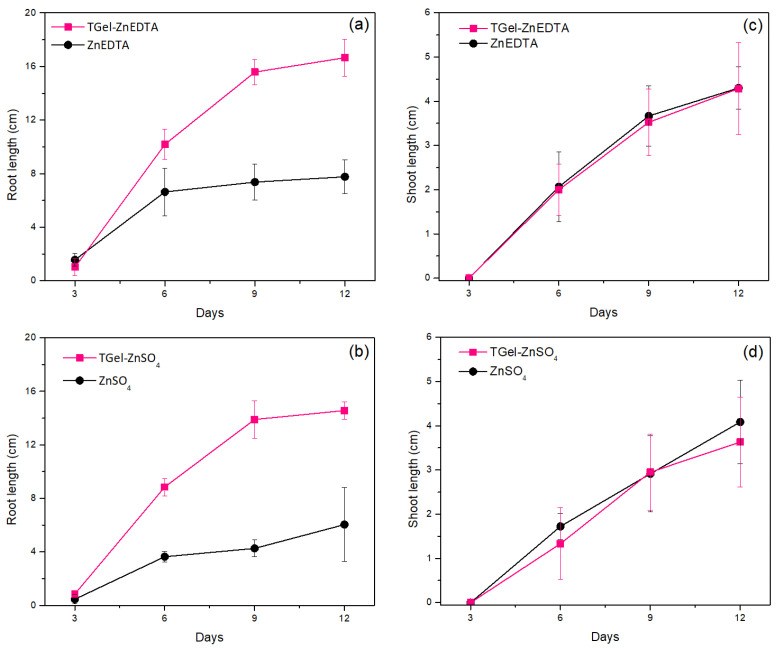
Evolution of (**a**,**b**) root length and (**c**,**d**) shoot length, as a function of time, up to 12 days after seed priming using the Zn-loaded TGels and the Zn control solutions. The data are shown as mean ± standard error.

Figure 6 shows the effects of Zn-loaded TGel nanopriming on cucumber seedling growth after germination, compared with the control Zn treatments, in terms of the evolution of the root and shoot lengths over time. Regardless of the type of polymeric gel, root growth was considerably higher for seed nanopriming with TGel-ZnEDTA and TGel-ZnSO_4_ compared with the use of the control ZnEDTA and ZnSO_4_ solutions (Figure 6a,b). After 6 days, the plants from seeds nanoprimed with the Zn-loaded TGels showed substantially higher root growth, which could be attributed to the effective uptake (by diffusion) of the loaded TGel throughout the seed tissues and the influence of subsequent Zn release on the germination process. In contrast, the seeds treated with the Zn controls showed apparent stabilization of root growth after 6 days, which could be explained by the high amounts of Zn available during seed priming using the control solutions, leading to sub-optimal utilization of the nutrients by the seeds. It should be noted that for all the seed treatments (Zn-loaded TGels, ZnEDTA, and ZnSO_4_ control solutions), the concentration of the zinc source was the same (100 mg L^−1^). Hence, the results for the root lengths indicated that the Zn-loaded TGels provided better accessibility of Zn for plant uptake compared with the control solutions. For the shoot lengths, the results for both TGel-ZnEDTA and TGel-ZnSO_4_ were close to those for the corresponding Zn control solutions (Figure 6c,d).

### 2.3. Zn Distribution and Photosynthetic Efficacy

**Figure 7:**
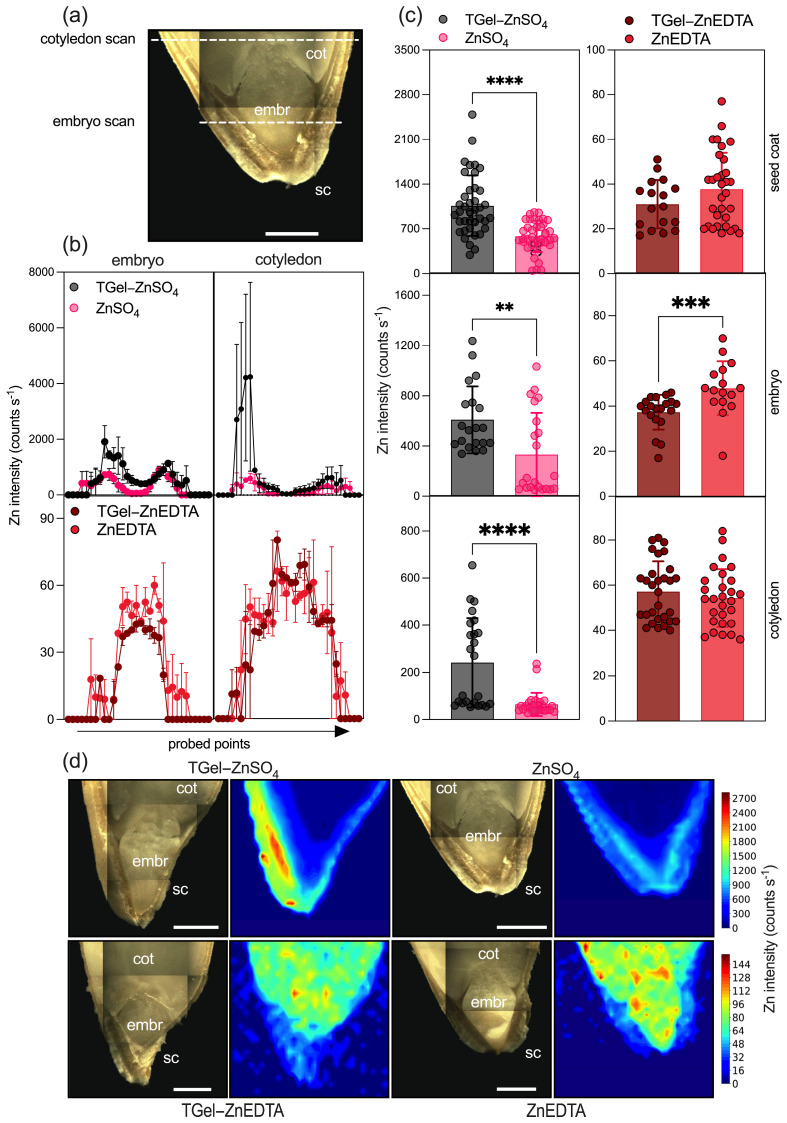
µ-XRF scanning of Zn distributions in cross-sections of cucumber seeds primed for 24 h with the Zn-loaded TGels or the ZnSO_4_ and ZnEDTA solutions at a Zn concentration of 100 mg L^−1^. (**a**) Photograph showing the scanned regions (indicated by the white dashed lines). (**b**) Zn intensities at the probed points in the embryo and cotyledon, with the data shown as mean ± maximum/minimum range. (**c**) Zn intensities obtained for the seed tissues (seed coat, embryo, and cotyledon) for the different treatments, where the bars show the mean ± standard deviation values for measurements using two independent biological replicates. Statistical analysis employed the Mann–Whitney *t*-test (*p* < 0.05). (**d**) Two-dimensional maps for the seed coat (sc), embryo (embr), and cotyledon (cot), with the highest Zn intensities obtained for the tissues exposed to the TGel-ZnSO_4_ formulation. Scale bars: 1 mm. The *p*-values lower or equal to 0.01, 0.001, and 0.0001 are represented with two, three, or four asterisks, respectively.

To evaluate the effects of the Zn-loaded TGel formulations on the absorption of Zn and its distribution in the cucumber seeds, specimens submitted to priming for 24 h were analyzed using micro X-ray fluorescence spectroscopy (μ-XRF). Figure 7 shows the Zn intensities recorded in scans of the embryo and cotyledon (Figure 7a), revealing higher Zn intensities for the seeds exposed to TGel-ZnSO_4_ compared with treatment with the control solution (Figure 7b). This was confirmed by tissue-based comparisons showing that the Zn intensities were significantly higher for the tissues (seed coat, embryo, and cotyledon) of seeds primed with TGel-ZnSO_4_ compared with those exposed to the ZnSO_4_ solution (Figure 7c). Regardless of the sulfate-based treatment, the Zn intensities were in the order of coating > embryo > cotyledon, as shown by the 2D maps (Figure 7d and Figure S1). Similar trends have been observed elsewhere for cucumber seeds treated with amine–epoxide and polyetheramine–epoxide gels with embedded micronutrients such as Fe^3+^ [23,24], as well as for soybean seeds exposed to ZnSO_4_ and bulk or nanosized ZnO solutions [25,26].

Conversely, no clear pattern was observed for the ZnEDTA-based treatments, where the inclusion of Zn in the gel led to virtually no differences in the Zn intensities for the seed coat and cotyledon tissues, while different intensities were found for the embryo. In addition, the Zn intensities for the cotyledon and embryo tissues were significantly higher than for the seed coat (details of this comparison are provided in Figure S2). Interestingly, the Zn intensities were around 10-fold lower than found for the sulfate-based treatments, suggesting either that there was lower adhesion of EDTA-chelated Zn to the cucumber seeds or that the EDTA-chelated Zn was readily absorbed towards the inner seed tissues (cotyledon and embryo), but at a slow rate, as observed elsewhere for soybean roots [27].

Our findings (by μ-XRF analysis) indicate that loaded TGel with different Zn sources may significantly influence the permeability and diffusion dynamics of metal across the perisperm–endosperm envelope of the seed, thereby enhancing the germination process. The effects observed after seed priming with Zn-loaded TGel formulations can be related to the cells’ osmotic equilibrium and membrane structure maintenance in which lignin present in seed coat cells is hydrophobic and acts as a natural barrier. The polyether backbone of TGel, composed of polyoxypropylene (-CH(CH_3_)-O-) units, enables potential interactions between the gel’s functional groups and the lignin-rich phase, influencing the transport of the solute (polymeric gel) to the seed coat surface and facilitating the internalization of loaded polymeric chains. The absorbed polymeric gel remains accessible due to possible interactions such as hydrogen bonding, cation-π, and hydrophobic interactions between TGel and the seed. The underlying mechanism and interactions involving amine–epoxide-based polymeric gels and seeds were recently demonstrated for the first time, as reported in reference [24].

The higher Zn uptake by seeds treated with sulfate-loaded TGel aligns with theoretical insights into Zn^2^⁺ cation coordination by functional groups within the polymeric gel network, as demonstrated by NOVC method calculations (Figure 3). The optimized Polymer_Frag_····Zn^2^⁺ geometries reveal orbital interactions in close proximity to nitrogen from amine groups and oxygen from polyether chains, establishing Zn^2^⁺ binding sites within the TGel framework. Given that the TGel structure comprises amide groups bonded to polyoxypropylene chains (three amides and three polyoxypropylene chains per structural unit), a high Zn^2^⁺ encapsulation capacity is suggested. The Polymer_Frag_····Zn^2^⁺ interactions, which exhibit partial covalent character, likely facilitate Zn^2^⁺ entrapment within the cross-linked polymer matrix. The correlation between theoretical analysis and μ-XRF results supports the potential of TGel as an efficient nutrient delivery system, enhancing the uptake of hydrophilic ionic zinc by seeds.

**Figure 8:**
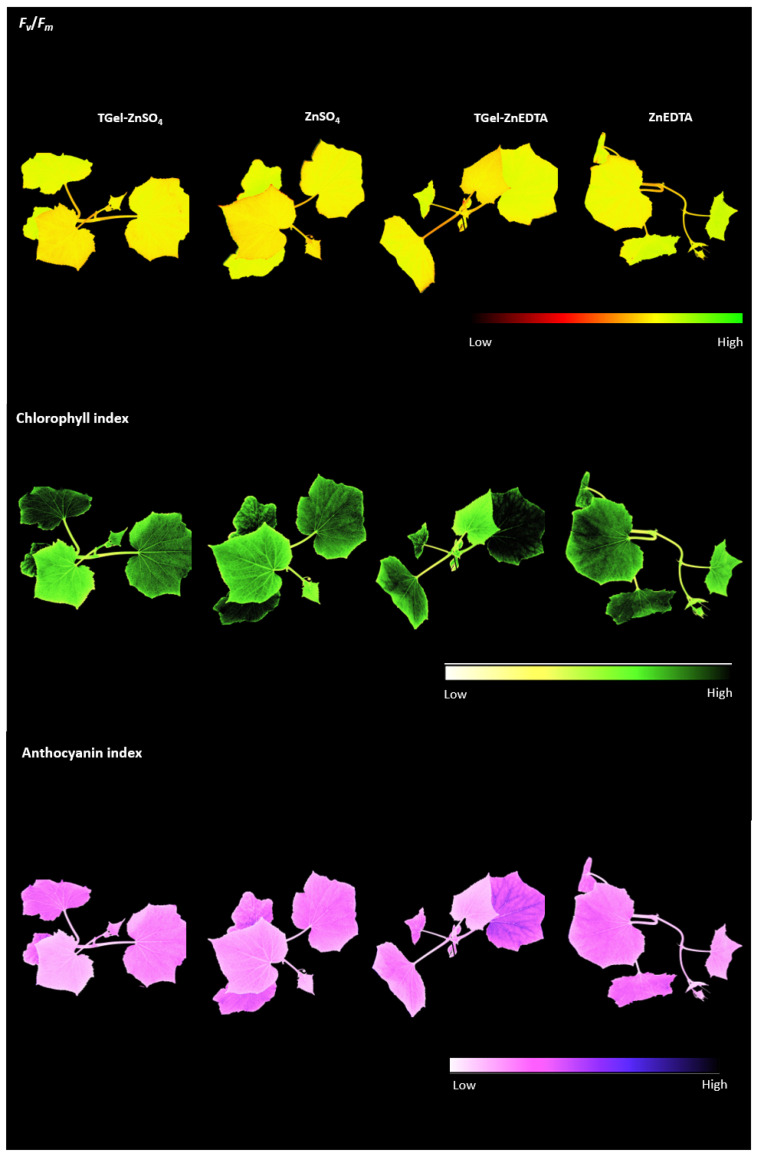
Multispectral images of the *C. sativus* plants after 18 days of growth for the seed treatments using the Zn-loaded TGels and the control Zn solutions. The plants were maintained in a hydroponic medium (Hoagland solution, without Zn). The values for the physiological parameter indices (PSII (*F_v_*/*F_m_*), chlorophyll *a*, and anthocyanin) are provided in the Supplementary Materials (Table S1).

In the seed priming assays, a high Zn concentration (100 mg L^−1^) was used to facilitate the evaluation of the effectiveness of the Zn-loaded TGel formulations as systems to improve Zn uptake by seeds and enhance the subsequent germination process. The seeds primed with the control Zn solutions showed fairly rapid initial root growth (day 6), followed by stabilization (days 9 and 12). The use of the Zn-loaded polymeric gels resulted in substantially faster root development without any negative effects from the seed treatment on the processes of germination and plant growth (Figure 5 and Figure 6). In addition, seed priming with the Zn-loaded TGels resulted in higher zinc concentrations in the shoots of the cucumber plants, with increases from 72 mg kg^−1^ (ZnSO_4_ control) to 92 mg kg^−1^ (TGel-ZnSO_4_) and from 56 mg kg^−1^ (ZnEDTA control) to 78 mg kg^−1^ (TGel-ZnEDTA) (Table 2). The observed increase in Zn uptake by seeds, followed by the enhanced metal concentration in cucumber plant shoots (as discussed above), demonstrates that the synthesized TGel effectively coordinates ionic zinc or Zn-EDTA complexes. The TGel characteristics enhance the polymer particles’ diffusion ability to penetrate the seed coat barrier, facilitating controlled zinc release and uptake. An evaluation of the safety of using the TGel formulations in agricultural applications, without any negative effects on plant development, was conducted by determining photosynthesis parameters for the cucumber plants on day 18. The treatments using the Zn-loaded TGels and the Zn control solutions (all at the same Zn concentration of 100 mg L^−1^) led to similar PSII activity, chlorophyll *a*, and anthocyanin indices (Figure 8, Table 2). The results showed that the use of the TGels to deliver Zn did not affect the physiological parameters of the plants. These findings revealed, for the first time, that the amine–polyether-based polymeric gels could (i) act as carrier systems to improve Zn absorption by seeds (as shown in the µ-XRF studies) and (ii) have a positive effect on root growth following the translocation of Zn during seedling development. The effects of the Zn-loaded TGels in potentiating plant development were significant since Zn plays important roles in improving cell integrity and stability, as well as in increasing root growth and acting as a defense factor against fungal infections [28,29]. Since positively charged nanoparticles tend to show greater adherence to the surfaces of soil particles and root tissues, which are both negatively charged [30], the use of the positively charged amine–epoxide particles as functional carriers opens possibilities for soil applications to combat fungal infections in cultivations such as corn and soybean, among others.

## 3. Conclusions

The use of polymer-based materials can have major effects on plants, as shown here for the seed priming formulations. The application of polymeric gels to improve seedling development represents a novel aspect of nanotechnology in agriculture. Seed priming with amine–epoxide particles based on TGel loaded with different zinc sources was shown to have positive effects on cucumber root growth and the absorption/translocation of Zn through the plant. Analysis of the Polymer_Frag_····[Zn(EDTA)]^2−^ complex showed that the Zn^2+^ cation interacted with the EDTA molecule by means of O····Zn noncovalent bonds, while the polymer fragment formed O–H····O· hydrogen bonds with the EDTA molecule. In contrast, in the Polymer_Frag_····Zn^2+^ complex, the Zn^2+^ cation directly interacted with the oxygen atoms of the polymer fragment by σ-bonds. Comprehensive μ-XRF analysis demonstrated that the levels of Zn were significantly higher in the tissues (seed coat, cotyledon, and embryo) of seeds primed with TGel-ZnSO_4_ compared with those exposed to the ZnSO_4_ control solution. Regardless of the Zn-loaded TGel treatment, the amounts of the micronutrient present in the cucumber shoots were higher than those obtained with the corresponding control solutions, with no negative effects on the physiological parameters of the plants. The experimental and theoretical findings of this study provide new perspectives on the use of polymeric nanomaterials as a green and safe seed priming technology. The insights from this work point towards the potential benefits that may be obtained from the application of polymeric nanogels in agriculture.

## 4. Materials and Methods

### 4.1. Chemicals

Diepoxy poly(ethylene glycol) (DPEG, C_3_H_5_O_2_-(C_2_H_4_O)_n_-C_3_H_5_O, average Mw = 500 g mol^−1^, CAS number: 26403-72-5), disodium zinc ethylenediaminetetraacetate hydrate (Zn-EDTA), zinc sulfate heptahydrate (ZnSO_4_.(H_2_O)_7_) and triamine-terminated polypropylene glycol (PPG)-based (known as Jeffamine T-403, M_w_ = 440 g mol^−1^, CAS number: 39423-51-3) were purchased from Sigma-Aldrich (São Paulo, Brazil). Jeffamine T-403 is a polyetheramine characterized by a backbone composed of repeating oxypropylene units. The amine groups are positioned on secondary carbon atoms at the end of aliphatic polyether chains. All reagents were used as received. Ultrapure water with a resistivity of 18.2 MΩ.cm was used in the synthesis of the polymer gels.

### 4.2. Preparation of Bisepoxide-Polyoxypropylenetriamine Gels: Unloaded and Zn-Loaded Polymeric Systems Chemicals

Amine–epoxide gels were synthesized according to a “click” reaction employing Jeffamine T-403 and DPEG, as described elsewhere [23,24,31], with adaptations. The initial step was the reaction of Jeffamine T-403 with DPEG in an aqueous medium (water) at a monomer concentration of 15 wt.% (mass of monomers in relation to the volume of water), ensuring complete solubilization of the reaction mixture. The molar ratio of amine to epoxide was maintained at 1:1 (Jeffamine T-403:DPEG). The 15 wt.% monomer solution was kept at 65 °C for 15 min to ensure initiation of the reaction. The solution was then diluted to 1.0 wt.% and allowed to react for a further 30 min. The final amine–epoxide solution was purified by dialysis in ultrapure water using regenerated cellulose membranes (denoted as TGel). In the next step, ZnSO_4_.(H_2_O)_7_ or Zn-EDTA solutions (100 mg L^−1^ of Zn) were individually mixed with 50 mg of TGel, lyophilized to ensure embedding of the Zn into the polymeric structure, and rehydrated with ultrapure water. The resulting Zn-loaded gels were denoted TGel-ZnSO_4_ and TGel-ZnEDTA.

### 4.3. Characterization of TGels

Determination of the hydrodynamic diameter (Dh), polydispersity index (PdI), and zeta potential (ζ) of the unloaded and Zn-loaded TGels employed a ZSU3100 Zetasizer Lab Blue analyzer (Malvern Instruments, Malvern, Worcestershire, UK) equipped with an OBIS solid-state laser source emitting at a wavelength of 633 nm. The measurements were performed at room temperature (~25 °C), in triplicate, with the results expressed as mean ± standard deviation (SD) (n ≥ 3). The morphologies of the gels were evaluated by transmission electron microscopy (TEM) using a JEM 100CXII instrument (JEOL, Peabody, MA, USA) operating at 100 kV. For acquisition of the images, a small droplet of the aqueous formulation was deposited onto a carbon-coated copper grid. The sample was then allowed to dry at room temperature in a desiccator to prevent contamination and ensure proper film formation. The polymeric gel used for the characterization was based on the solution diluted to 1.0 wt.%, denoted as TGel (see details in Section 4.2).

### 4.4. Computational Methods

All the molecular geometries were optimized without applying any geometric constraints. The vibrational frequency calculations were performed using the BP86 functional [32] and Grimme’s D3(BJ) dispersion corrections, with Becke–Johnson damping [21]. The Def2-TZVP basis set was used for all the calculations [33]. To improve computational efficiency, the RIJCOSX approximation was employed, with Coulomb integrals handled using the RI-J [34] method and the Def2/J auxiliary basis set [35]. To ensure that each optimized geometry corresponded to a true energy minimum, vibrational frequency analysis was performed to confirm the absence of imaginary frequencies, ensuring the accuracy of the computational model. All the calculations were carried out using the ORCA software package v. 5.0.4 [36]. The choice of the BP86-D3(BJ)/Def2-TZVP level of theory for geometry optimization was consistent with recommendations reported in the literature for systems involving noncovalent interactions [37]. The chemical bonding mechanism was investigated using the EDA-NOCV methodology [22]. These calculations were carried out with Amsterdam Density Functional (ADF 2021.1) software [38], employing the BP86-D3(BJ) theory level and the TZ2P basis set [39]. Scalar relativistic corrections were applied in a self-consistent manner using the zero-order regular approximation (ZORA) [40]. The ZORA-BP86-D3(BJ)/TZ2P computational model has been shown to be effective in clarifying the bonding mechanisms in systems involving noncovalent interactions [41]. Table S1 shows the optimized Cartesian coordinates for the compounds analyzed in this study.

The NOCV method allows orbital interactions between interacting fragments, such as, for example, Polymer_Frag_ and [Zn(EDTA)]^2−^, to be decomposed into pairwise contributions of the most relevant molecular orbitals. The deformation density, Δ*ρ*(r), the density differences of Polymer_Frag_ and [Zn(EDTA)]^2−^ before and after the chemical bond establishment, can be constructed from pairs of complementary eigenfunctions *ψ*_k_ and *ψ*_–k_ with eigenvalues *ν*_k_ and *ν*_–k_, respectively [22]:

(2) $$\Delta \rho \left(r\right)=\sum_{k}\nu_{k}\left[-\psi_{-k}^{2}\left(r\right)+\psi_{k}^{2}\left(r\right)\right]=\sum_{k}\Delta \rho_{k}\left(r\right)$$

This equation allows one to define the total charge deformation Δ*ρ*(r), created from the bond development, in terms of pairwise charge influences Δ*ρ*_k_(r), which originated from specific pairs of NOCV orbitals. The total orbital interaction energy Δ*E*_oi_ can be obtained from pairwise orbital interaction energies $\Delta E_{oi}^{k}$ that are related to Δ*ρ*_k_(r):

(3) $$\Delta E_{oi}=\sum_{k}\Delta E_{oi}^{k}=\sum_{k}\nu_{k}\left[-F_{-k,-k}^{TS}+F_{k,k}^{TS}\right]$$

The components $F_{-k,-k}^{TS}$ and $F_{k,k}^{TS}$ are diagonal transition state (TS) Kohn–Sham matrix elements. The TS expression accounts for the charge density, which is intermediary between the final complex density, Polymer_Frag_–[Zn(EDTA)]^2−^, and the superimposed moiety densities of Polymer_Frag_ and [Zn(EDTA)]^2−^. The $\Delta E_{oi}^{k}$ term of a specific bond can be visualized from the deformation density shape, Δ*ρ*_k_(r).

### 4.5. Seed Treatment

Seeds of cucumber (*Cucumis sativus*) were subjected to surface sterilization by sequential washes in 2% sodium hypochlorite (100 mL), followed by rinsing in deionized water (100 mL). The seeds were primed by application of the TGels loaded with the Zn sources (Zn-EDTA or ZnSO_4_), with the corresponding Zn solutions used as controls, to evaluate the effects on subsequent growth of the cucumber plants (considering the shoot and root lengths) following seed germination. The seeds were immersed in flasks (3 seeds/mL of suspension, with a total of 21 seeds) containing the TGel-ZnSO_4_ or TGel-ZnEDTA gel particles, with the Zn solutions as controls, allowing the flasks to stand in darkness at room temperature for 24 h, before germination on Petri dishes. The seeds primed with the Zn-loaded TGels or Zn solutions were arranged on filter papers in Petri dishes that were sealed to prevent water loss. For all seed assays, TGel formulations (both loaded and unloaded) were prepared based on the diluted system at a concentration of 1.0 wt.%, denoted as TGel. The zinc (Zn) concentration was maintained at 100 mg L⁻¹ for both control and Zn-loaded samples.

### 4.6. Micro X-Ray Fluorescence (μ-XRF) Analysis

The spatial distributions of Zn in the treated cucumber seed tissues were evaluated by micro X-ray fluorescence spectroscopy (µ-XRF), according to the procedure described elsewhere [24,42]. Briefly, the seeds treated for 24 h with TGel-ZnSO_4_, TGel-ZnEDTA, or the positive controls (ZnSO_4_ and ZnEDTA solutions) at a Zn concentration of 100 mg L^−1^ were cut transversely, and cross-sections of the medial regions were fixed on sections of 6 µm thickness polypropylene film (FPPP25-R3, VHG, Manchester, NH, USA), mounted on XRF sample vials (no. 1530, Chemplex, Palm City, FL, USA). The samples were loaded into an XRF spectrometer (Orbis PC, EDAX, Mahwah, NJ, USA), and the seed cross-sections were investigated by 32-point line scanning and 800-pixel two-dimensional mapping, encompassing the seed coat, embryo, and cotyledon tissues. The samples were exposed to a 30 µm polycapillary-focused X-ray beam set at 45 kV and 500 µA, with a 250 µm thickness Al primary filter. The spectra were acquired using a 30 mm^2^ silicon drift detector (SDD) with a dead time smaller than 10%. The dwell times were 15 s point^−1^ and 1 s pixel^−1^ for the line scans and maps, respectively. The analyses were carried out using two independent biological replicates, considering only the elemental intensities above the instrumental limit of detection (LOD) as valid, calculated as described in Equation (1) below. The intensities recorded for the seed coat, embryo, and cotyledon tissues were collected from the line scan data and were compared, as a function of the treatments, using the Mann–Whitney *t*-test at a 95% confidence level (*p* < 0.05). The data were processed using Prism v. 9.2.0 software (GraphPad, v. 10.1.1, Boston, MA, USA) and the Python-based (v. 3.9.6) Matplotlib library [43].

$$LOD=3\;\cdot \;\sqrt{\frac{BG}{t}}$$

where BG (cps) represents the average background counting rate, determined either from 10 randomly selected background points in elemental maps or from individual background values recorded along line scans. The acquisition time (t) is given in seconds (s).

### 4.7. Quantification of Zn in Cucumber Tissues, Using Energy-Dispersive X-Ray Fluorescence Spectroscopy (EDXRF) and Plant Photosynthesis Assessment

The concentrations of Zn in the cucumber shoot tissues were determined by EDXRF, as described by Montanha et al. [44], with minor modifications. Briefly, 100 mg portions of the dried and finely ground tissue samples were added to 6.3 mm diameter X-ray sample cups (SamplePrep no. 3577, SPEX, Livonia, MI, USA) sealed at the base with 6 μm thickness polypropylene film (FPPP25-R3, VHG, Manchester, NH, USA), gently pressed with a glass stick to remove void spaces, and covered with a spatula tip quantity of boric acid (Synth, Diadema, São Paulo, Brazil). The EDXRF analyses employed a spectrometer (model EDX-720, Shimadzu, Tokyo, Japan) with a 3 mm X-ray beam generated by a Rh anode-based X-ray tube at 50 kV and auto-tunable current up to 20% of the detector dead-time. The analyses were carried out under vacuum, and the acquisition time was 150 s. The X-ray spectra were recorded using a Si(Li) detector. The Zn Kα intensities were normalized by the Compton scattering intensities, and Zn quantification was based on an external calibration curve utilizing a set of plant-certified reference materials (Figure S3). All the analyses were performed with at least two independent biological replicates.

High-resolution images (2448 × 2448 pixels) of the plants were acquired to determine the maximum quantum efficiency of photosystem II (PSII) (*F_v_*/*F_m_*, where *F_m_* is maximum fluorescence and *F_v_* is variable fluorescence). Prior to the measurement, the plants were dark-adapted for 30 min and then illuminated with a 0.8 s pulse of saturating light (6320 μmol m^−2^ s^−1^). Next, the plants were submitted to actinic light for 5 min for the determination of non-photochemical quenching (NPQ = (F_m_ − F_m_’)/F_m_). Chlorophyll *a* fluorescence was measured using the excitation/emission combination of 620/730 nm. Multispectral images were also acquired to calculate the chlorophyll and anthocyanin indices [45,46]. Details of the procedures for obtaining these parameters can be found elsewhere [47]. All the parameters were obtained using a SeedReporter™ instrument (Enkhuizen, NL)and proprietary software (v. 5.5.1) (PhenoVation B.V., Wageningen, The Netherlands).

For these experiments (Zn quantification and photosynthesis studies), seeds were primed with the Zn-loaded TGels and the Zn control solutions (as described in Section 4.5). Following a 5-day germination period, uniform seedlings were identified and transferred to presterilized plastic pots (0.5 L) containing simulated Hoagland solution (without Zn, since this micronutrient was used in the seed priming treatments). After the 18-day growth period, the photosynthetic efficacy and the amounts of Zn in the plant shoots were determined as described above.

### 4.8. Statistical Analysis

GraphPad Prism v. 10.1.1 (GraphPad Software Inc., Boston, MA, USA) and OriginLab (v. 2022b, Northampton, MA, USA) were used for plotting graphs and performing statistical analyses. Evaluation of differences employed analysis of variance (ANOVA) combined with the Tukey test (*p* = 0.05). The results are shown as mean values in the tables and figures.

## Figures and Tables

**Table 1:** Investigation of the bonding scenarios: (i) Polymer_Frag_····Zn^2+^, (ii) [Polymer_Frag_(EDTA)]^4−^····Zn^2+^, and (iii) Polymer_Frag_····[Zn(EDTA)]^2−^, employing the EDA-NOCV methodology. The energy values are reported in kcal mol^−1^.

Interaction	Δ*E*_int_	Δ*V*_elstat_	Δ*E*_Pauli_	Δ*E*_oi_	Δ*E*_disp_	Δ*E*_oi,1_	Δ*E*_oi,2_	Δ*E*_oi,3_	Δ*E*_oi,4_	Δ*E*_oi,5_	Δ*E*_oi,6_
Polymer_Frag_····Zn^2+^	−415.6	−252.3	111.8	−257.7	−17.4	−67.0	−28.6	−25.6	−21.7	−11.1	−9.7
[Polymer_Frag_(EDTA)]^4−^····Zn^2+^	−1003.2	−822.2	114.5	−280.9	−14.6	−67.8	−27.7	−25.6	−24.6	−16.2	−13.4
Polymer_Frag_····[Zn(EDTA)]^2−^	−65.1	−68.0	64.7	−47.1	−14.7	−16.1	−8.8	–	–	–	–

Polymer_Frag_ refers to a fundamental repeating unit within the TGel polymer network. It represents a structural fragment that contains the key functional groups characteristic of the TGel polymer, playing a crucial role in defining its overall architecture and properties. Δ*E*_int_ (interaction energy), Δ*V*_elstat_ (electrostatic energy), Δ*E*_Pauli_ (Pauli repulsion energy), Δ*E*_oi_ (orbital interaction energy), and Δ*E*_disp_ (dispersion energy) correspond to the bonds analyzed as follows: Δ*E*_int_ = Δ*V*_elstat_ + Δ*E*_Pauli_ + Δ*E*_oi_ + Δ*E*_disp_, while Δ*E*_oi1–6_ represent the energetic contributions of each deformation density channel (Δρ_1–6_) associated with Δ*E*_oi_.

**Table 2:** Zn concentration on cucumber shoot tissues from plants whose seeds were primed with TGel-based or pure ZnSO_4_ and ZnEDTA solutions at 100 mg L^−1^ determined by EDXRF. Data from EDXRF represent the mean ± standard deviation of two independent biological replicates and were not subjected to any statistical comparison. Photosynthetic parameters *F_v_*/*F_m_*, chlorophyll *a* and anthocyanin indices for 18-day growth cucumber plants. No statistical differences were observed for five independent biological replicates. Identical letters, regardless of case (uppercase or lowercase), indicate that there is no statistical significant difference between the data for the corresponding treatment.

	EDXRF Analysis		Photosynthetic Parameters					
Zn Source	Zn Amount Present in Shoot (mg Kg^−1^)		PSII *F_v_/F_m_*		Chlorophyll *a* Index		Anthocyanin Index	
	Loaded-TGel	Control	Loaded-TGel	Control	Loaded-TGel	Control	Loaded-TGel	Control
ZnSO_4_	91.74	72.01	0.642Aa	0.636Aa	1.972Aa	1.818Aa	2.325Aa	2.071Aa
ZnEDTA	78.05	56.11	0.644Aa	0.650Aa	1.956Aa	1.994Aa	2.328Aa	2.422Aa

## Data Availability

The original contributions presented in this study are included in the article/Supplementary Materials. Further inquiries can be directed to the corresponding author.

## Supplementary Materials

The following supporting information can be downloaded at https://www.mdpi.com/article/10.3390/gels11030167/s1, Figure S1: Two-dimensional XRF maps of the Zn spatial distribution in cross-sectioned cucumber seeds primed with the TGel-based or pure ZnSO_4_ and ZnEDTA solutions at 100 mg L^−1^ Zn for 24-h; Figure S2: XRF Zn intensities recorded at the seed coat, embryo, and cotyledon of cross-sectioned cucumber seeds primed with the TGel-based or pure ZnSO_4_ and ZnEDTA solutions at 100 mg L^−1^ Zn for 24-h; Figure S3: Certified reference material-based external calibration curve used for quantitative Zn determination through energy-dispersive X-ray fluorescence spectroscopy; Table S1: Optimized Cartesian coordinates for the compounds analyzed in this study using the BP86–D3(BJ)/Def2–TZVP computational model.
