# Supplementary material for: Nano-Enabled Seed Treatment Using Bisepoxide-Polyoxypropylenetriamine Polymeric Gel with Different Embedded Zinc Sources
Source: Gels. 2025 Feb 26;11(3):167. doi: 10.3390/gels11030167 (PMC11942506; doi:10.3390/gels11030167)
Supplement: Supplementary file 1 [file gels-11-00167-s001.zip › gels-3488284-supplementary.pdf]

ELECTRONIC SUPPLEMENTARY MATERIAL

Nano-enabled seed treatment using bisepoxide-polyoxypropylenetriamine polymeric gel with different embedded zinc sources

Felipe B. Alves[a], Adela S. Martin[a,b], Bruno A. Fico[a], Vanessa S. A. Silva[a], Renato P. Orenha[a], Renato L. T. Parreira[a], Heber E. Andrada[a], Gabriel Sgarbiero Montanha[c], Higor J. F. A. da Silva[c], Eduardo de Almeida[c], Hudson W. P. de Carvalho[c,d], Natália Chittolina[c], Clíssia B. Mastrangelo[c], Eduardo F. Molina[a]*

[a] *Universidade de Franca, Av. Dr. Armando Salles Oliveira 201, Franca, SP, 14404-600, Brazil*

[b] *Universidad Pública de Navarra, Av. Cataluña, 31006 Pamplona, Navarra, España*

[c] *Centro de Energia Nuclear na Agricultura, Universidade de São Paulo, Av.Centerário 303, Piracicaba, SP, 13400-970, Brazil*

[d] *Chair of Soil Science, Mohammed VI Polytechnic University, Lot 660, Ben Guerir 43150, Morocco.*

*Correspondence: eduardo.molina@unifran.edu.br or molina_ferreira@yahoo.com.br

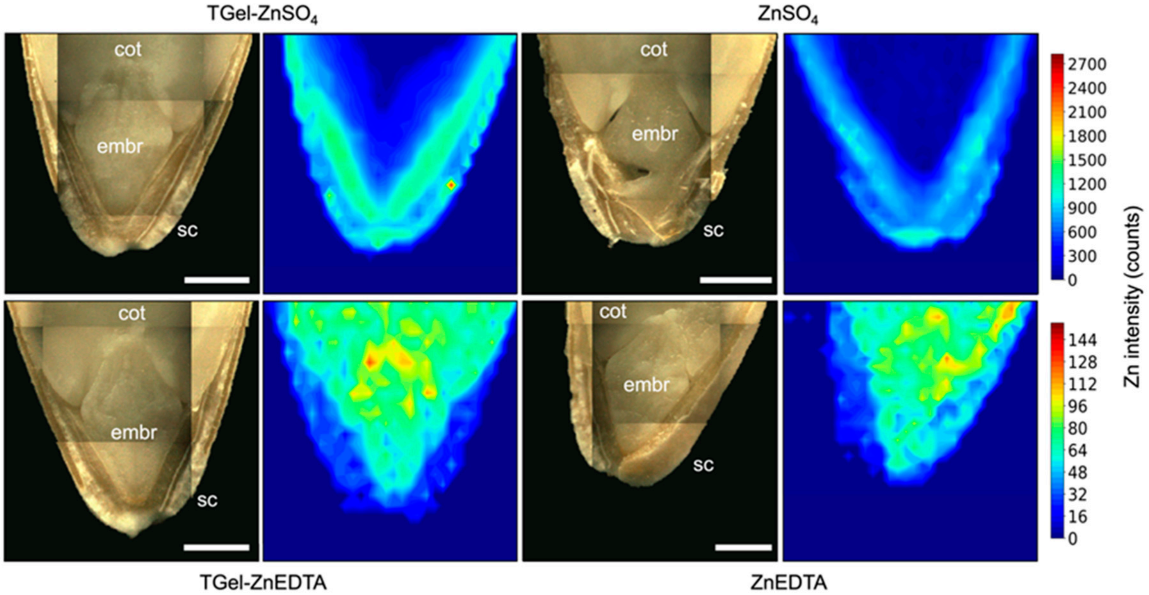

Figure S1. Two-dimensional XRF maps of the Zn spatial distribution in cross-sectioned cucumber seeds primed with the TGel-based or pure $ZnSO_4$ and ZnEDTA solutions at 100 mg L^{-1} Zn for 24-h. Data from an independent biological replicate. sc: seed coat; embr: embryo; cot: cotyledon. Scale: 1 mm.

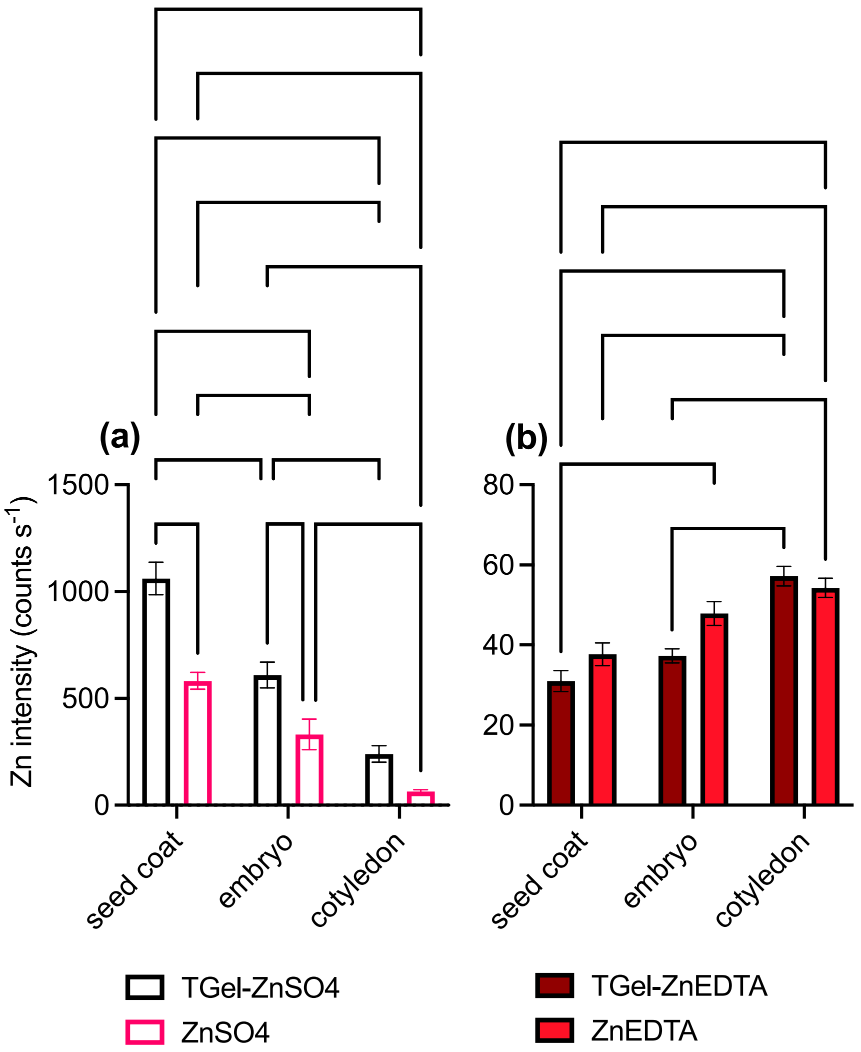

Figure S2. XRF Zn intensities recorded at the seed coat, embryo, and cotyledon of cross-sectioned cucumber seeds primed with the TGel-based or pure $ZnSO_4$ and ZnEDTA solutions at 100 mg L^{-1} Zn for 24-h. The data represents the mean ± standard error of data obtained on at least two independent biological replicates. Asterisks indicates statistical significance according to a two-way analysis of variance (where seed tissues and Zn sources were listed as factors) followed by Tukey's test at 0.05 level. The *p*-values lower or equal 0.05, 0.01, 0.001, and 0.0001 are represented with one, two, three, or four asterisks, respectively.

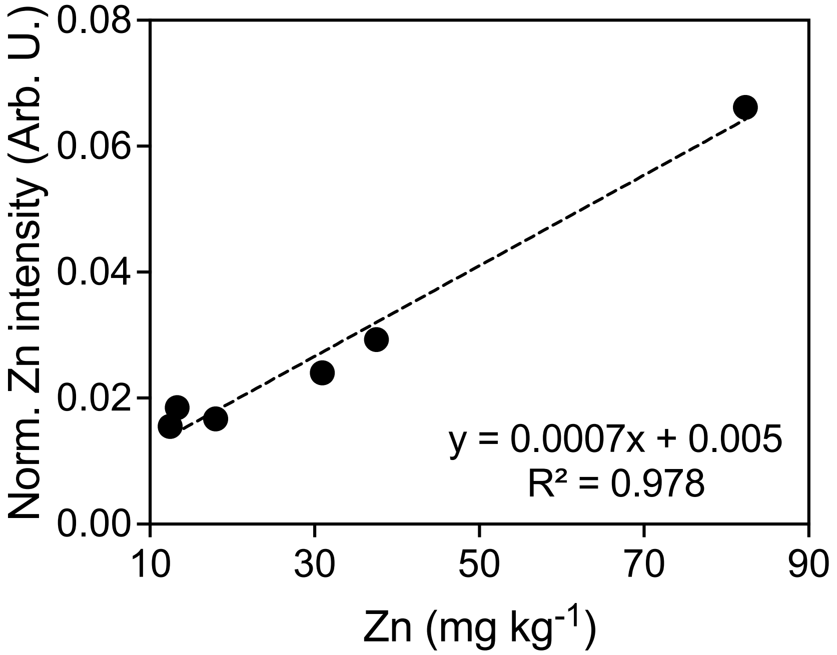

Figure S3. Certified reference material-based external calibration curve used for quantitative Zn determination through energy-dispersive X-ray fluorescence spectroscopy.

Table S1. Optimized Cartesian coordinates for the compounds analyzed in this study using the BP86–D3(BJ)/Def2–TZVP computational model.

Polymer$_{Frag}$····Zn^{2+}

C	-0.323459000	-5.170120000	2.380878000
C	-0.920892000	-4.063864000	1.520344000
C	1.480500000	-0.967122000	-2.160246000
C	-1.324979000	-2.871987000	2.378171000
C	3.608755000	-0.053757000	0.683209000
C	3.942892000	1.308799000	1.239382000
C	2.565673000	2.918098000	2.484489000
C	1.151810000	2.879849000	1.830362000
C	1.088356000	3.430230000	0.393751000
C	1.172081000	-2.207057000	-2.974974000
C	-1.716967000	-1.237370000	0.685578000
C	-0.523449000	3.331214000	-1.572112000
C	-1.631954000	2.471220000	-2.214202000
C	-2.713878000	-0.168766000	0.251468000
C	-2.613798000	1.800031000	-1.241684000
C	2.278959000	-1.270944000	-0.911514000
H	-0.761623000	-0.703288000	1.022737000
H	-0.235978000	0.185906000	-2.368460000
H	-3.213990000	0.273194000	1.126607000
H	-3.468268000	-0.575579000	-0.437837000
H	-1.859008000	-3.227181000	3.267215000
H	2.010317000	-0.208311000	-2.758779000
H	-3.104359000	2.557218000	-0.602949000
H	-3.401083000	1.288828000	-1.818120000
H	-0.414645000	-2.331332000	2.703341000
H	-2.228091000	3.145554000	-2.848302000
H	-1.573557000	1.301245000	-3.836532000
H	-1.070777000	-5.574129000	3.077198000
H	0.019365000	-6.004934000	1.752477000
H	0.337551000	3.351328000	-2.252433000
H	0.580779000	-1.952842000	-3.864214000
H	-0.882087000	2.920770000	0.387624000
H	0.412799000	-4.391727000	0.110340000
H	1.979413000	3.167288000	-0.195118000
H	3.276736000	-1.635805000	-1.193799000
H	1.770117000	-2.027838000	-0.289674000

H	-1.818153000	-4.434873000	0.992724000
H	2.485194000	3.144387000	3.554052000
H	3.211360000	3.671184000	2.010943000
H	0.534280000	-4.799606000	2.960315000
H	0.610225000	-2.932426000	-2.372036000
H	4.995356000	1.301652000	1.562504000
H	3.828006000	2.083796000	0.466203000
H	-1.408700000	-1.884163000	-0.149103000
H	3.478333000	-0.785131000	1.497988000
H	4.449246000	-0.383052000	0.052365000
H	2.102840000	-2.676065000	-3.323592000
N	-0.087975000	2.804442000	-0.254320000
O	0.737299000	1.475986000	1.854391000
O	3.129811000	1.604002000	2.400767000
O	0.235782000	-0.364863000	-1.671836000
O	-1.000392000	1.480845000	-3.069428000
O	-1.920260000	0.829514000	-0.436035000
O	-2.235660000	-1.937385000	1.768637000
O	0.045568000	-3.595239000	0.533978000
O	2.414476000	-0.032229000	-0.154775000
H	-0.883476000	4.365600000	-1.457037000
H	1.017133000	4.529216000	0.430122000
H	0.429806000	3.424700000	2.456044000
H	1.580389000	1.030608000	2.186635000
Zn	0.250647000	0.761238000	-0.028211000

Polymer$_{Frag}$····[Zn(EDTA)]$^{2–}$

N	2.491562000	0.437500000	1.332990000
C	1.142461000	0.866224000	0.984327000
H	1.187898000	1.533275000	0.113862000
H	0.636828000	1.415406000	1.800855000
C	0.255113000	-0.316591000	0.555196000
O	-0.987515000	-0.054108000	0.409426000
O	0.762566000	-1.451286000	0.357331000
C	2.593831000	-0.163249000	2.666617000
H	2.924023000	0.577020000	3.419240000
N	4.730237000	-0.205832000	-0.465780000
C	5.676074000	-1.323528000	-0.553633000
H	5.866401000	-1.687894000	0.466807000
H	6.636139000	-1.035986000	-1.019638000

C	5.082939000	-2.532072000	-1.345802000
O	5.889260000	-3.267007000	-1.938298000
O	3.802285000	-2.681390000	-1.292018000
C	4.543694000	0.505414000	-1.736511000
H	5.153776000	1.426828000	-1.776579000
H	4.881674000	-0.156152000	-2.548023000
C	3.067585000	0.866323000	-2.064498000
O	2.183799000	0.054692000	-1.615775000
O	2.869743000	1.888699000	-2.751103000
C	3.562182000	1.389885000	1.036066000
H	3.246380000	2.014908000	0.190095000
H	3.748184000	2.069227000	1.894107000
C	4.883819000	0.685014000	0.682420000
H	5.649879000	1.468203000	0.493844000
H	5.225220000	0.084087000	1.536298000
C	3.515960000	-1.412444000	2.751693000
O	4.034628000	-1.649775000	3.858430000
O	3.622817000	-2.105965000	1.678399000
H	1.593383000	-0.514173000	2.960490000
Zn	2.836472000	-1.342856000	-0.114444000
C	1.659831000	4.478956000	-0.780104000
C	0.254964000	5.051838000	-0.559197000
C	-1.287621000	-2.658646000	-1.833673000
C	-0.593877000	4.804190000	-1.809551000
C	-3.859572000	-4.328696000	-1.022356000
C	-3.619721000	-4.065868000	0.459636000
C	-4.933529000	-3.075485000	2.247992000
C	-5.487126000	-1.687159000	1.898096000
C	-4.485418000	-0.812809000	1.154797000
C	-0.415860000	-1.902842000	-2.835892000
C	-2.971926000	4.762874000	-1.380032000
C	-4.158514000	1.334745000	0.065965000
C	-3.169532000	2.090971000	0.975159000
C	-3.384213000	5.203009000	0.029197000
C	-3.780160000	3.366960000	1.552123000
C	-2.040455000	-3.787760000	-2.526955000
H	-3.801075000	4.935390000	-2.088264000
H	-1.771325000	-1.178295000	-0.609203000
H	-3.872428000	6.192169000	-0.011371000
H	-2.478893000	5.283017000	0.651438000

H	-0.018378000	5.148506000	-2.684709000
H	-0.637139000	-3.092826000	-1.053031000
H	-4.626235000	3.052285000	2.183524000
H	-3.028760000	3.874773000	2.184079000
H	-0.759314000	3.718113000	-1.928276000
H	-2.962262000	1.446843000	1.852975000
H	-1.464965000	1.447324000	0.274367000
H	2.219741000	5.064400000	-1.525684000
H	2.212796000	4.501195000	0.168723000
H	-3.572616000	0.737191000	-0.658112000
H	-4.748792000	2.068858000	-0.502517000
H	0.122813000	-1.080905000	-2.345668000
H	-5.938184000	0.345091000	0.363382000
H	-0.811532000	3.702847000	0.480753000
H	-3.586695000	-0.680314000	1.779978000
H	-4.146142000	-1.327209000	0.232906000
H	-1.329913000	-4.370799000	-3.131266000
H	-2.798818000	-3.345929000	-3.204789000
H	-5.793906000	-1.171415000	2.823934000
H	0.325972000	6.146175000	-0.415316000
H	-5.612281000	-3.561980000	2.968016000
H	-3.931282000	-3.001228000	2.702008000
H	-6.458045000	-2.585422000	0.507388000
H	1.633505000	3.443268000	-1.152824000
H	-1.037350000	-1.483253000	-3.643675000
H	-3.092834000	-4.924897000	0.912669000
H	-3.003614000	-3.162331000	0.569152000
H	-2.744227000	3.686852000	-1.360927000
H	-4.262011000	-3.415321000	-1.491153000
H	-4.606510000	-5.138594000	-1.122953000
H	0.346821000	-2.564440000	-3.274716000
N	-5.064164000	0.502263000	0.868617000
O	-6.693069000	-1.861232000	1.125421000
O	-4.911639000	-3.908874000	1.084349000
O	-2.260375000	-1.797834000	-1.226487000
O	-1.939854000	2.351772000	0.296654000
O	-4.332057000	4.298198000	0.614117000
O	-1.844290000	5.505230000	-1.856346000
O	-0.332020000	4.557312000	0.647047000
O	-2.666214000	-4.754700000	-1.669179000
